# Antimicrobial stewardship: Assessment of knowledge, awareness of antimicrobial resistance and appropriate antibiotic use among healthcare students in a Nigerian University

**DOI:** 10.1186/s12909-021-02912-4

**Published:** 2021-09-10

**Authors:** Wuraola Akande-Sholabi, Amen T. Ajamu

**Affiliations:** Department of Clinical Pharmacy and Pharmacy Administration, Faculty of Pharmacy, University of Ibadan, Ibadan, Nigeria

**Keywords:** Antibiotic use, Antimicrobial resistance, Antimicrobial stewardship, Healthcare students, Knowledge

## Abstract

**Background:**

Inappropriate use of antibiotics is a major cause of antimicrobial resistance (AMR). Inadequate knowledge about AMR among healthcare students could affect their practice of antimicrobial stewardship as future healthcare professionals. This study aims to assess the use of antibiotics and knowledge of AMR among future healthcare professionals of a Nigerian University.

**Methods:**

Respondents’ knowledge of antimicrobial resistance, use of antibiotics, and source of antibiotics in the past 12 months was explored using a self-administered questionnaire. Multivariate analyses were used to evaluate the relationship between specific variables and respondents’ knowledge.

**Results:**

Of the 939 questionnaires administered to the students, 866 were filled given a response rate of 92.2 %. A total of (765; 88.3 %) of the respondents were aware that antimicrobial resistance makes it harder to eliminate the infection from the body as existing drugs become less effective. In all 824, (95.2 %) of the respondents had use antibiotics in the past 12 months. The use of antibiotics to treat malaria was self-reported by (175; 21.2 %). About half (432; 52.4 %) purchased the antibiotics from community pharmacies, while others obtained their antibiotics from the hospitals (192; 23.3 %), patent medicine stores (150; 18.2 %), and friends and family (50; 6.1 %) in the last 12 months. In all 506, (58.4 %) had good knowledge of antimicrobial resistance. Logistic regression shows that students in 3rd to 6th year 9.29 [AOR = 9.29, 95 % CI: (3.7–22.96)], had greater knowledge of antimicrobial resistance.

**Conclusions:**

The healthcare students demonstrated a moderate knowledge of AMR. This underscores the need to adopt several educational tactics to introduce the concepts of AMR to the students and ensure there are strict policies to regulate the flow of antibiotics.

**Supplementary Information:**

The online version contains supplementary material available at 10.1186/s12909-021-02912-4.

## Introduction

The advent of antibiotics indicated that previously untreatable medical conditions became treatable, surgeries became largely successful, and the entire medical practice experience has not been the usual since then [1]. Unfortunately, this admirable phase of medical practice is being threatened by the inappropriate use of antibiotics, leading to antimicrobial resistance (AMR). It has been reported that inappropriate use of antibiotics is estimated to be 20–50 % in Nigeria [2]. The term “inappropriate use” comprises the use of antibiotics to treat all forms of infections, incomplete dosage regimen during treatment, and sharing of antibiotics with others without prescription by a physician [3]. Furthermore, antimicrobial resistance (AMR) occurs naturally when microorganisms (such as bacteria, fungi, viruses, and parasites) can not be killed by an antimicrobial drug to which they were hitherto sensitive, usually due to genetic mutations [4].

Globally, antimicrobial resistance is a foremost public health challenge, particularly in developing countries. Misuse of antimicrobials is assisted in developing countries by their availability over the counter, without a prescription, and through unregulated supply chains [5, 6]. AMR leads to decreased medication efficiency, complication during treatment of patients, or an increase in management cost. There is, therefore, an urgent need to tackle antimicrobial resistance in developing countries specifically in Africa because it is being ignored and yet vastly associated with high morbidity and mortality [7].

Healthcare students as future prescribers and dispensers of antimicrobials have a crucial role in reducing the inappropriate use of antibiotics; there has been a rising volume of research reports in the literature concentrated on exploring the knowledge, attitude, and practice of students towards antibiotics use. Previous studies conducted among health care students showed an existing gap between knowledge and practice and reported the need for educational interventions to improve knowledge and awareness of antibiotic stewardship among healthcare students [8, 9]. It is worthy to note that, the way an individual perceives an idea may also affect his/her behavior on an issue, thus generating behavioral and contextual barriers. Evidence suggests that strategic actions such as antimicrobial stewardship must be enforced. Therefore, improving the knowledge of healthcare students especially pharmacy students is crucial in the fight against AMR. Well-educated pharmacists can contribute to the decrease of irrational use of antimicrobials. Thus, making the pharmacist’s role in the healthcare system of developing countries significant in the global battle against AMR [10].

Efforts channeled towards decreasing the occurrence of antimicrobial resistance are not essentially about restricting access to an antibiotic, but to ensure that antibiotics are used appropriately. This means that accurate and effective antibiotic stewardship should be directed at rational use and not just access restriction. Moreover, the World Health Organization reports encourage the training of healthcare students on appropriate antimicrobial prescribing and an introduction of the concepts of antimicrobial stewardship into the undergraduate programme [11].

It is worthy of note that in Nigeria, most of the previous studies on inappropriate use of antibiotics and knowledge on antimicrobial resistance were focused on general populations [12, 13], with a paucity of information on healthcare students. Thus, this study aims to evaluate the use of antibiotics and knowledge of antimicrobial resistance among future healthcare professionals at a Nigerian University.

## Method

### Study design and settings

This is a university-based cross-sectional study using a self-administered questionnaire. It was conducted among undergraduate students of the Faculty of Pharmacy, Department of Nursing, and Department of Medicine and Surgery, between August and November 2019. Presently in Nigeria, the Bachelor of Pharmacy and Bachelor of Nursing degree are a 5-year programme, while the Bachelor of Medicine and Surgery degree is a 6-year programme. Eligible participants were registered undergraduate Pharmacy, Nursing, and Medicine and Surgery students for the 2018/2019 academic session and consented to partake in the study. Students that were absent and non-consenting were excluded from the study.

### Sample size determination

Based on the population of 1521 registered students obtained from the faculty management, Pharmacy (360), Medicine and Surgery (881), and Nursing (280) the confidence level was set at 95 % and the alpha error at 5 %, arriving at a sample size of 316 using Yamane’s formula [14]. The target sample size arrived after calculation using Yamane’s formula was Pharmacy (75), Medicine and Surgery (183), and Nursing (58). Additionally, to cater for the possibility of a low response rate, which is not uncommon among students, an attrition rate close to 200 % was used.

### Sampling and data collection procedure

At each level in each faculty, a compulsory course for the students was identified. The study respondents were consecutively approached shortly after the mandatory course. The researcher informed the participants of the study objectives. Questionnaires were disseminated to all consenting healthcare students physically and recovered within 20–25 min. Study participation was voluntary and the students were informed of the possibility of withdrawing from the study anytime. Response anonymity and confidentiality were reiterated to the respondents. Measures were put in place to prevent multiple filling of the questionnaire by the respondents.

### Data collection instrument

The questionnaire used for the study was designed by the researchers after a thorough review of similar studies [7, 8], as well as utilizing researchers’ proficiency. The questionnaire comprised three sections and a total of 20 questions. Section A comprised of demographic characteristics of the students. Section B consists of questions on participants’ use of antibiotics, frequency of antibiotic use, and source of antibiotics in the past 12 months. Section C comprises of 10-item questions with a 5-point Likert scale response option ranging from strongly agree (5) to strongly disagree (1) to evaluate students’ knowledge of antimicrobial resistance. The overall score for the knowledge questions was categorized into ‘good’ and ‘poor’ depending on respondents’ scores in each domain. For the 10-item statements on knowledge with a 5-point Likert scale response, a total score of at least 40 (≥ 80 %) out of the maximum obtainable score of 50 was categorized as ‘good’ knowledge, while a knowledge score < 40 (< 80 %) was categorized as ‘poor’ knowledge. The binary categorization of knowledge scores was adapted from Bloom’s cut-off criteria and other similar studies [15–17]. There were four negatively worded knowledge questions (items 2, 3, 9, 10), these questions were reversed scored.

### Pretest and content validation

Content validity of the questionnaire was conducted by one pharmacist and one medical doctor in academia, to establish the inclusiveness of question-items when compared with the objectives of the study. Thereafter, face validity was established by administering the questionnaire to thirty students from the Pharmacy, Nursing, and Medicine and Surgery Department to ascertain the ease of comprehension of questions or statements in the questionnaire. These students were exempted from the main study and the response from the students led to some modifications including reformatting some questions to eliminate response ambiguity.

### Statistical analysis

Data were coded, cleaned, and analyzed using the IBM Statistical Package for Social Sciences (version 23). Descriptive statistics such as frequency counts and percentages, mean, and standard deviation were used to summarize and present the result. Prior to the analysis, all negatively worded items in the knowledge questions were reversed scored. Chi-square test was used to investigate the association between relevant demographic characteristics and the level of knowledge on antimicrobial resistance and proper handling of antibiotics. Logistic regression analysis was used to explore relationships between significant variables and knowledge of antimicrobial resistance. The level of significance was set at *P* < 0.05.

### Ethics approval

 Ethics approval for the study was obtained from the joint University of Ibadan/University College Hospital Institution Review Board with approval number UI/EC/19/0403.

## Results

Table 1 shows details on the demographic characteristics of the study population. Of the 939 questionnaires administered to the students, 866 were filled given a response rate of 92.2 %. Females were (447; 51.6 %) and males were (419; 48.4 %). The half of respondents were medical students (436; 50.3 %) while nursing was the least represented (155; 17.9 %).

**Table 1:** Demographic characteristics of healthcare students (*n* = 866)

Variable	Number of students	Percentage
**Age**		
$\le$ 20	405	46.8
21–29	453	52.3
$\ge$ 30	8	0.9
**Gender**		
Male	419	48.4
Female	447	51.6
**Department and level of Study**		
**Nursing** ***n = 155***		
Year 1	32	20.6
Year 2	28	18.1
Year 3	39	25.2
Year 4	35	22.6
Year 5	21	13.5
**Pharmacy** ***n = 275***		
Year 1	46	16.7
Year 2	22	8.0
Year 3	44	16.0
Year 4	88	32.0
Year 5	75	27.3
**Medicine and surgery** ***n*** = 436		
Year 1	47	10.8
Year 2	79	18.1
Year 3	104	23.9
Year 4	64	14.7
Year 5	88	20.2
Year 6	54	12.4

The assessment of respondents’ knowledge of antimicrobial resistance is shown in Table 2. Overall, a total of 506 (58.4 %) had good knowledge of antimicrobial resistance and proper handling of antibiotics.

**Table 2:** Respondents’ knowledge on antimicrobial resistance (*n* = 866)

	Variable	SA		A		U	D	SD
		**n (%)**		**n (%)**		**n (%)**	**n (%)**	**n (%)**
1.	Antimicrobial resistance is the ability of microbes to grow in the presence of a chemical (drug) that would normally kill them or limit their growth	407 (47.0)		381 (44.0)		53(6.1)	13 (1.5)	12 (1.4)
2.	It is not necessary to complete the regimen of an antibiotic to reduce the chances of the occurrence of bacteria resistance to drug	32 (3.7)		69 (8.0)		83(9.6)	282(32.6)	400 (46.2)
3.	It is not necessary to use the correct dose of an antibiotic to reduce the chances of the occurrence of bacteria resistance to drug	25 (2.9)		52 (6.0)		87(10.0)	284(32.8)	418 (48.3)
4.	Antimicrobial resistance cause death	213 (24.6)		337 (38.9)		225(26.0)	58 (6.7)	33 (3.8)
5.	Improper self-medication can cause antimicrobial resistance	346 (40.0)		405 (46.8)		85 (9.8)	15 (1.7)	15 (1.7)
6.	Antimicrobial resistance affects all age groups	334 (38.6)		405 (46.8)		102(11.8)	18 (2.1)	7(0.8)
7.	Antimicrobial resistance makes it harder to eliminate infections from the body as existing drugs become less effective	401 (46.3)		364 (42.0)		77 (8.9)	16 (1.8)	8 (0.9)
8.	Antimicrobial resistance can lead to spread of infections due to ineffectiveness of standard treatment	347 (40.1)		409 (47.2)		87 (10.0)	14 (1.6)	9 (1.0)
9.	Antibiotics will improve the outcome of the treatment of common cold	92 (10.6)		260 (30.0)		241(27.8)	166(19.2)	107 (12.4)
10.	Antibiotics will improve the outcome of the treatment of uncomplicated malaria	62 (7.2)		176 (20.3)		273(31.5)	202(23.3)	153 (17.7)
	**Cut-off score**		**Frequency n (%)**		**Remark**			
	< 8 (i.e., < 80 %)		360 (41.6)		Poor knowledge			
	≥ 8(i.e., ≥ 80 %)		506 (58.4)		Good knowledge			

Maximum obtainable score = 50; % individual score = score obtained by an individual ÷ by total obtainable score x 100. Strongly agree (SA) = 5, Agree (A) = 4, Undecided (U) = 3, Disagree(D) = 2, Strongly disagree (SD) = 1. Items 2, 3, 9, 10, were negatively worded know questions, these questions were reversed scored.

The classification of antibiotic use showed malaria (175; 16.9 %), cough (168; 16.3 %), and sore throat (164; 15.9 %) were the commonest ailments antibiotic was used for, while amoxicillin (265; 32.2 %), ciprofloxacin (166; 17.2 %) and metronidazole (157; 16.2 %) were the most common antibiotics used according to the frequency of use. More than half of the respondents (432; 52.4 %) purchased their antibiotics from the community pharmacies, others obtained their antibiotics from hospitals (192; 23.3 %) and patent medicine vendors (150; 18.2 %). Overall, a total of 824, (95.2 %) of respondents had received antibiotics in the last 12 months. Of the 824 students that self-reported the use of antibiotics, the majority 663, (76.6 %) used it only when needed and (104; 29.4 %) got drug information from patient information leaflets in the drugs Details in Table 3.

**Table 3:** Respondents’ classification of the use of antibiotics, frequency of antibiotic use, and source of antibiotics in the last 12 months (*n* = 824)

Variable	Number of students	Percentage
***Ailments ₼ (n = 1031)***		
Malaria Cough	175 168	16.9 16.3
Sore throat	164	15.9
Common cold	125	12.1
Typhoid	107	10.4
Genitourinary infections	96	9.3
Diarrhea Diseases of the skin	88 62	8.5 6.0
Ulcer	29	2.8
Sexually Transmitted diseases	17	1.8
***Antibiotics ₼ (n = 967)***		
Amoxicillin Ciprofloxacin Metronidazole	265 166 157	27.5 17.2 16.2
Amoxicillin + clavulanic acid	136	14.0
Tetracycline Erythromycin	95 63	9.8 6.5
Penicillin	37	3.8
Doxycycline	30	3.1
Azithromycin	18	1.9
***Source of Antibiotic (n = 824)***		
Community pharmacy	432	52.4
Hospital	192	23.3
Patent Medicine Vendor	150	18.2
Friends and family	50	6.1
***Frequency of use of Antibiotic ₼ (n = 866)***		
When needed	663	76.6
Once yearly	44	5.1
Once every 3 months	40	4.6
Monthly	22	2.5
Weekly	55	6.4
Never	42	4.8
***Source of drug information about dosage and duration of use of antibiotic (n = 354)***		
Patient information leaflet in the drug	104	29.4
Family and friends	67	18.9
Curriculum/classroom	60	16.9
Textbook	54	15.3
Internet (Medscape, google and others)	41	11.6
Print media (newspaper and Magazines)	15	4.2
Social media	13	3.7

₼ **=** multiple responses

The multivariate analysis for the variables that were significantly associated with good knowledge of antimicrobial resistance at the bivariate level showed that students in 3rd year [AOR = 1.75; 95 % CI 1.09–2.80], 4th year [AOR = 5.47; 95 % CI 3.13–9.55], 5th year [AOR = 5.42; 95 % CI 2.94–9.97], and 6th year [AOR = 9.29; 95 % CI 3.7–22.96], demonstrated greater knowledge of antimicrobial resistance (*p* < 0.05) as shown in Table 4.

**Table 4:** Predictors of Knowledge of Antimicrobial Resistance among healthcare students (*n* = 866)

Variables	Knowledge on Antimicrobial Resistance		Unadjusted Odd ratio		Adjusted Odd ratio	
			**OR (95 % CI)**	*p-value*	**AOR (95 % CI)**	*p-value*
	**Good** **n (%)**	**Poor** **n (%)**				
**Gender**						
Male	242 (57.8)	177 (42.2)	1			
Female	264 (59.1)	183 (40.9)	1.05 (0.80–1.38)	0697	1.28 (0.93–1.76)	0.117
**Age-group (years)**						
≤ 20 (ref)	200 (49.4)	205 (5.6)	1			
21–29	299 (66.0)	154 (34.0)	1.99 (1.51–2.62)	0.001*	0.78 (0.51–1.19)	0.261
≥ 30	7 (87.5)	1 (12.5)	7.17 (0.87–58.84)	0.006	2.47 (0.28–21.26)	0.409
**Department**						
Nursing ref	82 (52.9)	73 (47.1)	1			
Pharmacy	154 (56.0)	121 (44.0)	1.13 (0.76–1.68)	0.536	0.90 (0.57–1.40)	0.645
Medicine and Surgery	270 (61.9)	166 (38.1)	1.44 (1.00-2.09)	0.050	1.31 (0.86–2.01)	0.205
**Level of Education**						
Year 1	45 (36.0)	80 (64.0)	1		1	
Year 2	56 (43.4)	73 (56.6)	1.36 (0.82–2.26)	0.228	1.31 (0.78–2.19)	0.305
Year 3	95 (50.8)	92 (49.2)	1.84 (1.15–2.92)	0.010*	1.75 (1.09–2.80)	0.021*
Year 4	134 (71.7)	53 (28.3)	4.49 (2.77–7.29)	0.001*	5.47 (3.13–9.55)	0.001*
Year 5	131 (71.2)	53 (28.8)	4.39 (2.70–7.14)	0.001*	5.42 (2.94–9.97)	0.001*
Year 6 (ref)	45 (83.3)	9 (16.7)	3.98 (3.98–19.85)	0.001*	9.29 (3.75–22.96)	0.001*

CI Confidence Interval, *significance *p* value < 0.05

## Discussion

This study aimed to evaluate the use of antibiotics and knowledge of antimicrobial resistance among future healthcare professionals in a Nigerian University. Appropriate use of antimicrobials is central to curbing the incidence of antimicrobial resistance (AMR), which can be accomplished by changing prescribers’ behavior and knowledge [11]. In this study, it was shown that about 41.6 % of the participants demonstrated poor knowledge about antimicrobial resistance. This is lower compared to other studies reported in Portugal, Trinidad and Tobago, which reported a better understanding of antimicrobial resistance among the study participants [18, 19]. The difference in antimicrobial stewardship across countries might be attributed to the variations in demographic characteristics, questions used in the survey, and socioeconomic factors of the respondents [18, 19].

It is apparent that among the students who appear to be knowledgeable on antimicrobial resistance, the appropriate use of antibiotics is not revealed in their regular life as documented in this study. This is evident from the proportion of respondents who used antibiotics for the management of malaria, cough, sore throat, and the common cold. This inappropriate use of antibiotics might be due to a lack of information on antimicrobial use. A study conducted in Nigeria reported antibiotics to be among the top three medications used for self-medication among undergraduate healthcare students, who believed that they have acquired the requisite medical knowledge of what to use for a particular condition treated [20].

However, the majority of the participants showed an imperative characteristic to acquire their antibiotics from either the community pharmacy or hospital and use the antibiotics only when needed. They were also aware of the fact that antimicrobial resistance makes it harder to eliminate the infection from the body as existing drugs become less effective. Surprisingly, about a fifth of the respondents purchased their antibiotics from proprietary and patent medicine vendors (PPMV). In Nigeria, PPMVs are defined as “a person without formal training in pharmacy who sells orthodox pharmaceutical products on a retail basis for profit” [21]. Regulations permit PPMVs to sell a limited number of pre-packaged, over-the-counter medicines and medical products but prohibit them from selling prescription medications (including antibiotics) or conducting invasive medical procedures (e.g. injections) [21]. Even though the sale of antibiotics is outside PPMVs’ current scope of practice, some respondents were able to purchase this medication from them, this might be due to lack of strict and enforced laws and regulations on how antibiotics are prescribed and dispensed in retail pharmacies in Nigeria and among these vendors. It is worthy to note that the purchase of antibiotics without a prescription is common in Nigeria. Moreover, future healthcare professionals should be aware of the legal scope of practice of PPMVs allowed by the law. This result is similar to findings in Nigeria where 60 % of sick children received antibiotics from PPMVs [22]. These results suggest support courses either in the curriculum or as a workshop/seminar could be introduced to educate the students at the university level on inappropriate use and purchase of antibiotics and promote awareness of antimicrobial stewardship among future healthcare professionals. In addition, there is a possibility of overprescribing antibiotics without the performance of antimicrobial susceptibility test in retail pharmacies and among the vendors. It is important to emphasize the need for performing the antimicrobial susceptibility tests before prescribing antibiotics. Thus, medical students should be aware that prescribing antibiotics in a non-controlled manner is not a good practice.

The findings from this study show antibiotics use in the management of malaria, malaria is one of the major health risks in tropical regions with high morbidity and death rates. Antimalarial drugs are the recommended medications for the chemoprophylaxis or treatment of malaria and not antibiotics. However, some antibiotics (tetracyclines and macrolides) are effective against malaria parasites [23]. Nonetheless, the inappropriate use of antibiotics in treating malaria could be promoting the emergence of bacterial resistance. Besides, more than half of the participants reported using antibiotics for the management of cough, sore throat, and the common cold. A systematic review on antibiotics use for the common cold and acute purulent rhinitis reported there is no benefit from antibiotics for the common cold, sore throat, and cough [24]. Common cold, sore throat, and cough are mostly viral infections, and antibiotics fight bacteria and not a virus. These results are comparable to outcomes in another study conducted in Rwanda, where 27 % of the respondents had used antibiotics to treat common cold and sore throat [25].

It was observed in this study that the respondents’ year of study and advanced age were significantly associated with the level of their knowledge of antimicrobial resistance. This might be a consequence of better hands-on experience and a greater degree of exposure to clinical practice as the students advance in their year of learning. Programs like the Student Industrial Work Experience Scheme (SIWES) and ward rounds which begin after the third year of study might also be responsible for the improved level of knowledge. These findings suggest that there is a need for timeliness of the efforts targeted at ensuring public education on antimicrobial resistance. This becomes necessary to steadily improve students’ knowledge of antimicrobial stewardship, which will consequently lead to an increase in the value-added services to be offered by these future healthcare providers to the public. This will be especially useful in the context of patients’ counseling on the awareness of antimicrobial resistance. Thus, the need to create awareness about antimicrobial stewardship among healthcare professionals in Nigeria has been emphasized in a recent study [26]. Furthermore, reinforcement of antimicrobial stewardship in all health facilities would create persistent awareness on antimicrobial resistance to healthcare professionals [27, 28].

## Limitations

The results reported may be subject to recall bias, social appropriateness whereby the respondents either over or under-reported the use of antibiotics in the past 12 months. Furthermore, the inherent limitation of self-reporting may not be excluded. Future study may consider other possible socio-demographics such as living conditions and socio-economic status that may influence future healthcare professional knowledge and awareness in engaging in antimicrobial stewardship.

## Conclusions

The healthcare students demonstrated a moderate knowledge of AMR, and their knowledge significantly varies across year of study. Our findings suggest enhancing the students’ level of knowledge about the use of antibiotics might improve their antimicrobial stewardship. This study advocate for educational interventions to improve the knowledge and understanding of antimicrobial resistance among healthcare students and introducing the concept of AMR to healthcare students either via seminars/symposium or workshop starting at early years of study. Awareness campaign through media considering public health is also recommended. In addition, strict policies should be put in place to regulate the flow of antibiotics and to prevent the purchase of antibiotics without a medical prescription. This might be an approach to improve rational antimicrobial use and curb antimicrobial resistance.

## Supplementary information

**Additional file 1.**

## Data Availability

The datasets used and/or analysed during the current study are available from the corresponding author on reasonable request.

## Abbreviations

AMR: Antimicrobial Resistance
PPMV: Proprietary and Patent Medicine Vendors
SIWES: Student Industrial Work Experience Scheme
