# Supplementary material for: Antimicrobial stewardship: Assessment of knowledge, awareness of antimicrobial resistance and appropriate antibiotic use among healthcare students in a Nigerian University
Source: BMC Med Educ. 2021 Sep 10;21:488. doi: 10.1186/s12909-021-02912-4 (PMC8434738; doi:10.1186/s12909-021-02912-4)
Supplement: Supplementary file 1 — Additional file 1. [file 12909_2021_2912_MOESM1_ESM.docx]

**FACULTY OF PHARMACY**

**DEPARTMENT OF CLINICAL PHARMACY AND PHARMACY ADMINISTRATION**

**QUESTIONNAIRE ON KNOWLEDGE, AWARENESS OF ANTIMICROBIAL RESISTANCE AND ANTIBIOTIC USE AMONG HEALTHCARE STUDENTS AT THE UNIVERSITY OF IBADAN.**

Dear Respondent, this questionnaire is aimed at assessing the knowledge, awareness of antimicrobial resistance and antibiotic use.

Please note that every information provided in this questionnaire will be treated with confidentiality. Thank you for taking time to participate in this study

**Section A**: Demographic characteristics

Date ___________________________

Faculty: ____________________________

Level of study: 1^st^ Year ___ 2^nd^ Year __3^rd^ Year ___ 4^th^ Year ___5^th^ Year __6^th^ Year ___

1. Gender (a) Male _____ (b) Female ______
2. Age __________

**Section B**:

**Have you used antibiotics in the past 12 months? Tick the appropriate response as it applies to you**? Yes ____________No ________________

If “No” **skip** the remaining questions in this section

**For which of the following conditions have you used an antibiotic in the past 12months?**

**Please tick appropriate response**

- Malaria ____________
- Disease of the Skin _____________
- Sore throat ______________
- Common Cold ______________
- Cough _________________
- Genitourinary infections _________________
- Sexually Transmitted diseases
- Ulcer____________
- Others (specify)_______________________________________

**Which of the following antibiotics have you used in the last 12 months?**

- Amoxicillin____________
- Penicillin ___________
- Amoxicillin + clavulanic acid _____________
- Metronidazole _______________
- Azithromycin ________________
- Ciprofloxacin_____________________
- Doxycycline
- Tetracycline______________________
- Erythromycin_________________________
- others (specify) ___________________________

**Sources of drug information about the dosage and duration of use of the antibiotics?**

**please tick appropriate response**

- Social media _______
- Textbook __________
- Family and friends __________
- Curriculum/classroom_____________
- Internet (Medscape, google and others)
- Patient information leaflet in the drug __________
- Print media (newspaper and magazines) _____________

**How often do you use antibiotics, please tick appropriate response?**

- When needed_________
- Once yearly___________
- Once every 3 months ___________
- Monthly ______________
- Weekly _____________
- Never _______________

**Source of antibiotics please tick appropriate response**

- Community pharmacy ______________
- Friends/family____________
- Patent medicine vendor__________
- Hospital______________

**Section C: Knowledge on antimicrobial resistance and proper handling of antibiotics**

| **S/N** | **Question** | **Strongly Disagree** | **Disagree** | **Undecided** | **Agree** | **Strongly Agree** |
| --- | --- | --- | --- | --- | --- | --- |
| 1 | Antimicrobial resistance is the ability of microbes to grow in the presence of a chemical (drug) that would normally kill them or limit their growth |  |  |  |  |  |
| 2 | It is necessary to complete the regimen of an antibiotic to reduce the chances of the occurrence of bacteria resistance to drug |  |  |  |  |  |
| 3 | It is necessary to use the correct dose of an antibiotic to reduce the chances of the occurrence of bacteria resistance to drug |  |  |  |  |  |
| 4 | Antimicrobial resistance cause death |  |  |  |  |  |
| 5 | Improper self-medication can cause antimicrobial resistance |  |  |  |  |  |
| 6 | Antimicrobial resistance affects all age groups |  |  |  |  |  |
| 7 | Antimicrobial resistance makes it harder to eliminate infections from the body as existing drugs become less effective |  |  |  |  |  |
| 8 | Antimicrobial resistance can lead to spread of infections due to ineffectiveness of standard treatment |  |  |  |  |  |
| 9 | Antibiotics will improve the outcome of the treatment of common cold. |  |  |  |  |  |
| 10 | Antibiotics will improve the outcome of the treatment of uncomplicated malaria. |  |  |  |  |  |
